# Oysters and Typhoid in Paris

**Published:** 1913-04

**Authors:** 


OYSTERS AND TYPHOID IN PARIS. Paul Vincey, *Society of Mediciine of Paris,* February 7, 1913. For the period of 1906-1910, the average mortality per annum has been 9.2 per 100,000 population. Analysed, the mortality was 9.6 for males, 8.8 for females. It was less than 2 per 100,000 of the ages beyond 60, exfor a rise after 80, which is not significant on account of the small number of persons of advanced ages. The highest rate was for males of about 25, 20.2 per 100,000 of this age. In 1911, owing to the failure of the spring water normally supplied for domestic purposes and the use of Seine water (which is not bad according to American standards) the mortality was doubled. (Note—Typhoid is rarely seen in Paris hospitals, and about half the cases come from the suburbs which have a comparatively small population. Ed.) The author then compares the seasonal mortality with statistics of importation of oysters into the city. The latter are about as for American cities, practically nil for May-August, rising rapidly to a December maximum and dropping to about a third for January and February. The mortality rates for the same period (1906-10) were 13 for June and August (per 100,000 population), 19 for July and December, 20-25 for the other months. The morbidity rates were 122 for June, 126 for August, 132 for July, and varied from 131 to 205 for the other months. The average mortality was 21 and morbidity 159, indicating a nearly perfect reporting of cases. While in America, one would be inclined to ascribe the seasonal variations to greater tendency to drinking impure water, the author concludes that about a quarter of the deaths from typhoid in Paris are due to oysters. The objections to his conclusions were freely voiced in the discussion.

